# MetaFluAD: meta-learning for predicting antigenic distances among influenza viruses

**DOI:** 10.1093/bib/bbae395

**Published:** 2024-08-12

**Authors:** Qitao Jia, Yuanling Xia, Fanglin Dong, Weihua Li

**Affiliations:** School of Information Science and Engineering, Yunnan University, Kunming 650500, China; State Key Laboratory for Conservation and Utilization of Bio-Resources in Yunnan, Yunnan University, Kunming 650500, China; School of Information Science and Engineering, Yunnan University, Kunming 650500, China; School of Information Science and Engineering, Yunnan University, Kunming 650500, China

**Keywords:** influenza virus, antigenic distance, meta learning

## Abstract

Influenza viruses rapidly evolve to evade previously acquired human immunity. Maintaining vaccine efficacy necessitates continuous monitoring of antigenic differences among strains. Traditional serological methods for assessing these differences are labor-intensive and time-consuming, highlighting the need for efficient computational approaches. This paper proposes MetaFluAD, a meta-learning-based method designed to predict quantitative antigenic distances among strains. This method models antigenic relationships between strains, represented by their hemagglutinin (HA) sequences, as a weighted attributed network. Employing a graph neural network (GNN)-based encoder combined with a robust meta-learning framework, MetaFluAD learns comprehensive strain representations within a unified space encompassing both antigenic and genetic features. Furthermore, the meta-learning framework enables knowledge transfer across different influenza subtypes, allowing MetaFluAD to achieve remarkable performance with limited data. MetaFluAD demonstrates excellent performance and overall robustness across various influenza subtypes, including A/H3N2, A/H1N1, A/H5N1, B/Victoria, and B/Yamagata. MetaFluAD synthesizes the strengths of GNN-based encoding and meta-learning to offer a promising approach for accurate antigenic distance prediction. Additionally, MetaFluAD can effectively identify dominant antigenic clusters within seasonal influenza viruses, aiding in the development of effective vaccines and efficient monitoring of viral evolution.

## Introduction

Influenza viruses have the potential to cause both seasonal epidemics and pandemics, posing a significant threat to public health. Although vaccination remains the most effective means of mitigating this impact, the constant evolution of influenza viruses enables them to evade host immunity, thus necessitating frequent vaccine updates to maintain efficacy [1]. To select and update vaccine strains, the World Health Organization’s Global Influenza Surveillance and Response System (GISRS) tracks the genotypes and antigenic properties of circulating strains. The surface glycoprotein hemagglutinin (HA) is the primary locus of interaction with the human immune system; therefore, antigenic properties of influenza viruses are often assessed through hemagglutination inhibition (HI) assays, which measure the cross-reactivity of one virus strain to serum raised against another strain. However, such experiments are laborious, low-throughput, and unsuitable for early-stage screening. As a result, efficient computational methods for predicting antigenicity have become an alternative to traditional serological assays and are important for early screening of vaccines.

Compared with HI assays, computational methods can facilitate the analysis of antigenic properties on a large scale. Smith *et al*. [2] pioneered antigenic cartography, a computational approach that effectively visualizes antigenic distances and detects antigenic drift. Subsequent refinements by Cai *et al*. [3], Barnett *et al*. [4], and Sun *et al*. [5] have extended this work. However, these early methods relied solely on HI data, neglecting the rich information contained within genetic sequences. This limitation prompted Huang *et al*. [6] and Wang *et al*. [7] to integrate genetic information into HI data for antigenicity inference. While these methods offer a valuable framework for studying strain phenotypes, they often leverage low-rank matrix decomposition, which captures primarily shallow, linear features. Besides, these methods primarily integrate HA sequence similarity with antigenic data, potentially overlooking valuable insights from genomics.

Beyond antigenic cartography, research efforts have focused on modeling the relationship between HA sequence and antigenic dissimilarity using machine learning. These approaches seek to predict antigenic properties directly from genetic data, potentially enabling faster and more comprehensive vaccine screening. Early attempts utilized various machine learning algorithms, including regression models [8–10], naive Bayesian classifier [11], and random forest [12]. Nevertheless, these methods focused on the antigenicity-dominant positions[13], which determined the majority of antigenic phenotype, and relied heavily on the feature of antigenicity-dominant positions to represent the HA sequences. Although Lees *et al*. [14], Peng *et al*. [11], and Yao *et al*. [12] attempted to address this limitation, existing approaches remain bound by the inherent constraints of linear feature modeling in traditional machine learning.

Harnessing the remarkable ability of deep learning to model intricate nonlinear relationships, researchers are increasingly exploring its applications in antigenicity analysis. Studies like those by Lee *et al*. [15] and Forghani and Khachay [16] leveraged convolutional neural networks (CNNs) to predict the antigenicity of H3N2 and H1N1 subtypes, respectively. Yin *et al*. [17] further investigated influenza antigenicity prediction using a two-dimensional CNN model, representing sequences with ProtVec embeddings [18]. More recently, Xia *et al*. [19] encoded each amino acid into a one-hot vector, then coupled CNNs with bidirectional long-short-term memory neural networks to predict antigenicity. Similarly, Yin *et al*.[20] employed contrastive learning to improve antigenicity prediction. Meng *et al*. [21] employed CNNs to predict the antigenic relationship and further deduces antigenic clusters. However, these methods focused on qualitative antigenicity prediction. To address this, Peng *et al*. [22] proposed a novel method using attribute network embedding to compute quantitative antigenic distances in influenza A H3N2. While these advancements demonstrate the potential of deep learning to accelerate vaccine screening, their performance remains contingent upon the volume and quality of available data, as well as the ability of models to extract relevant features from these datasets effectively.

To address this issue, we propose a meta-learning-based method, MetaFluAD, to predict quantitative antigenic distances among strains. This paper models antigenic relationships between strains represented by their hemagglutinin (HA) sequences as a weighted attributed network, wherein edge weights reflect the degree of antigenic dissimilarity. Leveraging a graph neural network (GNN)-based encoder, MetaFluAD effectively learns strain representations within this unified space that encompasses both antigenic and genetic features. This facilitates efficient knowledge transfer across diverse influenza lineages via meta-learning, enabling accurate antigenic distance prediction even with limited data. Extensive evaluations demonstrate MetaFluAD’s superior performance and generalizability, offering a promising avenue for accurate antigenic distance prediction in influenza viruses, even when data are scarce.

## Materials and methods

### Datasets

To comprehensively evaluate the performance of MetaFluAD, we constructed four datasets called D_H3N2, D_H1N1, D_Vic, and D_Yam, encompassing diverse influenza subtypes or lineages from A/H3N2, A/H1N1, B/Victoria, and B/Yamagata. These datasets, curated from World Health Organization (WHO) annual reports spanning 2003 to 2023, include both strains and corresponding antigenic distances derived from hemagglutination inhibition assays.

The datasets download the HA sequences for the strains from EpiFlu Database [23, 24] and Influenza Research Database [25]. The datasets prioritized the longest HA sequence if a strain had multiple sequences. Next, sequences were aligned using MEGA [26].

The antigenic distance between strains $i$ and $j$ could be defined by Archetti–Horsfall distance [27] as follows: 


(1) \begin{align*}& d_{ij}=\sqrt{\frac{H_{ii} \times H_{jj}}{H_{ij} \times H_{ji}}},\end{align*}

where the HI titer $H_{ij}$ is the maximum dilution of antisera raised in strain $v_{i}$ to inhibit cell agglutination caused by strain $v_{j}$. A smaller value of ${d_{ij}}$ corresponds to higher antigenic similarity between the strains. Additionally, the shape space theory [28] shows the antigenic distance between strains is linearly related to the logarithmic of the HI measurement. And, the logarithmic transformation can help alleviate the problem of sparse data by compressing the range of values, reducing the variance of the data, and allowing better model performance. Thus, this paper uses $\log{d_{ij}}$ for model training and prediction.

To improve data quality and reliability, the incomplete or ambiguous titer values denoted by “<”, “>”, and ”ND” (Not Done) were excluded. Then duplicated entries were merged into single entries, utilizing their averages as the final HI record. Finally, for A/H3N2, A/H1N1, B/Victoria, and B/Yamagata, we obtained 64975, 68897, 17502, and 21141 titer data entries, respectively. According to equation 1, we calculated the antigenic distances as defined above.

Finally, the four datasets D_H3N2, D_H1N1, D_Vic, and D_Yam consist of 163, 84, 40, and 31 strains, respectively; they consist of 1102, 635, 201, and 168 pairwise antigenic distances, respectively. In addition to the aforementioned datasets, a dataset D_H5N1 [17] is also employed to evaluate the performance of the models, where D_H5N1 contains 260 A/H5N1 strains and 666 antigenic distances.

### Antigenic dissimilarity network

This paper employs a weighted attributed network to model the antigenic relatedness between the $n$ strains $\mathcal{V}=\{v_{1}, v_{2}, \ldots ,v_{n}\}$, called an antigenic dissimilarity network $\mathcal{G}=\mathcal{(V,X,E)}$, where each node denotes a stain $v_{i} \in \mathcal{V}$; $\mathcal{X}=\{x_{1},x_{2},\ldots ,x_{n}\}^{T}$ is the attribute matrix of nodes; $\mathcal{E}$ is the edge set. The topology structure of graph $\mathcal{G}$ can be denoted by an adjacency matrix $\mathbf{A}\in \mathbb{R}^{n \times n}$, where $a_{ij}=d_{ij} $ if $v_{i},v_{j} \in \mathcal{E}$, indicating the antigenic distance between strain $v_{i}$ and strain $v_{j}$.

The hemagglutinin (HA) protein, a crucial surface glycoprotein of influenza viruses, mediates viral entry into host cells by binding to sialic acid-containing receptors. The HA needs to be cleaved into two subunits, HA1 and HA2, by cellular proteases to be functional [1]. The HA1 subunit forms the globular head domain of HA, which encompasses the critical receptor binding site [29] responsible for host cells. Notably, HA1 exhibits a higher mutation rate compared with HA2, making it a key determinant of antigenic variability. Therefore, the HA1 sequence is used as the attribute of the node in an antigenic dissimilarity network. For each node $v \in \mathcal{V}$ representing a strain, its HA1 sequence is converted to a vector $x_{v}\in \mathbb{R}^{(l-2) \times 100}$ by Protvec [18], where $l$ is the length of the HA1 sequence. Algorithm 1 (see Supplementary Data) describes the main steps involved in constructing an antigenic dissimilarity network.

### Overview of MetaFluAD

**Figure 1:**
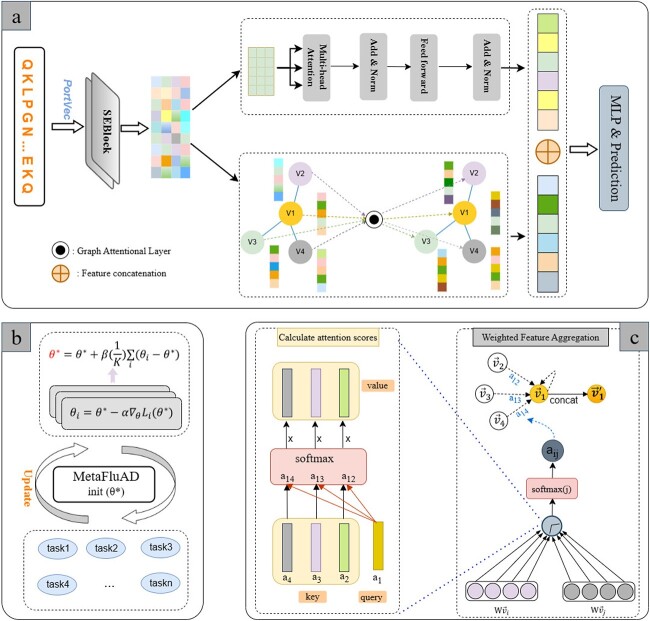
Overview of MetaFluAD; (a) MetaFluAD architecture: featuring SEBlock units, an embedding module and a multi-layer perception for antigenic distance prediction; (b) meta-learning of MetaFluAD: learning a well-generalized model from antigenic dissimilarity networks, which can be quickly adapted to antigenic distance prediction with limited samples; (c)additional details for the graph embedding module: it updates the representation of node by computing attention coefficients and weighting the features of the adjacent nodes.

Figure 1 overviews the basic idea and architecture of MetaFluAD. This figure highlights the deployment of a GNN-based embedding module combined with self-attention mechanisms to explore the relationships between strains and the final use of a multi-layer perception to calculate antigenic distances.

#### The architecture of MetaFluAD

GNNs are specifically designed to work with graph-structured data and are widely utilized in bioinformatics [30]. Squeeze-and-Excitation Networks [31] enhance model performance by adaptively recalibrating channel-wise feature responses, leading to more informative and discriminative representations with minimal additional computational cost. Thus, MetaFluAD strategically combines SEBlock and a GNN-based embedding module to extract and represent essential features of influenza strains. Then, a multi-layer perception is utilized to calculate antigenic distances.

The model initiates the process by engaging a SEBlock module to extract salient features from the ProtVec-encoded sequence representations $\mathbf{X}\in \mathbb{R}^{n \times (l-2)\times 100}$, transforming their initial sequence representation into $\mathbf{X}\in \mathbb{R}^{n \times 256}$, where $n$ represents the number of strains.

Within the GNN-based embedding module, we employ a graph attention network (GAT) [32], a specific type of GNN augmented with multi-head attention mechanisms, to delve into the intricate interrelations among the strains. Multi-head attention mechanisms allow the model to focus on different aspects of node relationships simultaneously. By assigning attention scores to edges, GAT effectively learns the importance of each neighbor for a specific node, leading to more informative representations of strains. In multi-head GAT, each head is represented by a learned weight matrix $W_{i}$, where $i$ is the index of the head. Each attention head calculates the attention score $e_{vu}^{h}$ between the node $v$ and its neighbor node $u \in \mathcal{N}(v)$ respectively, which can be expressed by the following formula: 


(2) \begin{align*}& e_{vu}^{h} = \text{LeakyReLU}\left(\vec{a}_{h} \cdot\left[W_{i}\vec{x}_{v}, W_{i} \vec{x}_{u}\right]\right)\end{align*}

The activation function LeakyReLU is defined as 


(3) \begin{align*}& \text{LeakyReLU}(x) = \begin{cases} x, & \text{if}\ x> 0 \\ \alpha x, & \text{otherwise} \end{cases}.\end{align*}

In addition, $\vec{a}_{h}$ is the attention parameter learned by head $h$; $\vec{x}_{v}$ and $\vec{x}_{u}$ represent strains $v$ and $u$, respectively. The attention score $e_{vu}^{h}$ is used to calculate the normalized attention weight $a_{vu}^{h}$: 


(4) \begin{align*}& \alpha_{vu}^{h} = \frac{\exp(e_{vu}^{h})}{\sum_{k \in \mathcal N(v)} \exp(e_{vk}^{h})},\end{align*}

where $\mathcal{N}(v)$ represents the set of neighbor strains of strain $v$. These attention weights are used to weigh and summarize the features of neighbor antigenic information. Finally, the final representation of the strain $v$ is obtained by combining the antigenic information: 


(5) \begin{align*} \vec x_{v}^{\prime} &= \sigma\Biggl(\text{Concat}\Biggl(\sum_{h=1}^{H} \sum_{u \in \mathcal N(v)} \alpha_{vu}^{h} W_{h} \vec x_{u}\Biggr)\Biggr),\end{align*}

where $H$ is the number of multi head, $\vec x_{v}^{\prime}$ is the final representation of strain $v$ after combining antigenic features, and $\sigma $ is the ReLU activation function. During training, the multi-head GAT employs attention weights, enabling each head to independently focus on specific aspects of strains. Subsequently, these head-specific representations are concatenated, generating an enriched encoding that unveils the intricacies of strain relationships. Following the multi-head GAT, we obtain the HA1 sequence feature matrix, denoted as $\mathbf{E}_{G}\in \mathbb{R}^{n \times 256}$, where $n$ represents the number of strains.

Smith *et al*.’s [2] observation that specific amino acid substitutions can have disproportionate effects on antigenicity underscores the likelihood of an uneven distribution of individual position contributions to antigenic variation. While GNNs offer powerful representation learning for strains, relying solely on them may inadvertently mask this crucial feature. Therefore, extracting imbalanced genetic information features from another perspective will be very beneficial to model performance. To this end, we leverage the multi-head self-attention mechanism, proven effective in the biomedical field [30, 33], to capture the position-specific importance of amino acids within HA1 sequences. For the $\mathbf{X}\in \mathbb{R}^{n \times 256}$ from SEBlock, the $i$th self-attention calculates is as follows: 


(6) \begin{align*}& \begin{aligned} &\mathbf{Q}_{i}= \mathbf{X}\mathbf{W}_{i}^{Q} \\ &\mathbf{K}_{i}= \mathbf{X}\mathbf{W}_{i}^{K} \\ &\mathbf{V}_{i}= \mathbf{X}\mathbf{W}_{i}^{V} \\ \mathbf{A}_{i} = &\text{softmax}\left(\frac{\mathbf{Q}_{i}\mathbf{K}_{i}^{T}}{\sqrt{D}}\right)\mathbf{V}_{i} ,\end{aligned}\end{align*}

where $D$ is the number of hidden units. The features $\mathbf{A}_{i}$ from head$_{i}$ are then concatenated and fed into a feed-forward neural network layer with two linear transformations and a nonlinear activation function. This network outputs the feature matrix $\mathbf{E}_{T} \in \mathbb{R}^{n \times 256}$, providing a finely tuned feature representation for each strain.

In our computational framework, the GAT-based block and the self-attention block process the input data in parallel. Each block yields an output feature matrix with a dimensionality of 256. To amalgamate the learned representations from both blocks, we concatenate the two matrices $\mathbf{E}_{G}$ and $\mathbf{E}_{T}$ along the feature dimension, resulting in a comprehensive strain embedding matrix $\mathbf{E} \in \mathbb{R}^{n \times 512}$, which serves as a potent foundation for downstream tasks such as clustering and antigenic distance prediction.

Based on the embeddings of strains $\mathbf{E}\in \mathbb{R}^{n \times 512}$, a 1024-dimensional vector $e_{ij}$, concatenated from $e_{i}$ and $e_{j}$ for a pair of strains $v_{i}$ and $v_{j}$, is fed into a multi-layer perceptron and predicts the antigenic distance: 


(7) \begin{align*}& \hat{d_{ij}} = \mathbf{W}_{2} \sigma(\mathbf{W}_{1} e_{ij} + \mathbf{b}_{1}) + b_{2},\end{align*}

where $\sigma $ represents leakyReLU activation function.

#### Meta-learning for predicting antigenic distances

Predicting antigenic distances between influenza viruses is crucial for assessing vaccine efficacy and preventing influenza. Owing to the laborious nature of serological techniques, experimentally derived antigenic distances tend to demonstrate sparsity, which substantially constrains the performance of antigenic distance computation models. To address this, MetaFluAD is specifically designed for antigenic distance prediction. Unlike existing methods, MetaFluAD leverages a two-phase approach, combining efficient meta-training on diverse subtype or lineage datasets with meta-testing on limited data from the target subtypes or lineages, enabling rapid adaptation and improved performance.

In the meta-learning phase, MetaFluAD employs the tasks derived from some subtype or lineage datasets as meta-tasks. Each meta-task is dedicated to predicting antigenic distances within its respective partition. By iteratively optimizing the model across these diverse tasks, MetaFluAD acquires a comprehensive understanding of the relationships among influenza viruses, thereby establishing robust initial model parameters.

To facilitate understanding, we introduce some notations. Let $T_{i}$ denote a task involving the prediction of antigenic distances for a specific influenza subtype or lineage. The total set of tasks is represented as $T= T^{t} \cup T^{s}$, where $T^{t}$ and $T^{s}$ are disjoint sets of tasks dedicated to meta-training and meta-testing, respectively. Furthermore, let $ \theta $ represent all the learnable parameters in MetaFluAD, which includes both the parameters in virus representation learning and prediction model.

Within the architecture of our MetaFluAD model, we employ the Reptile meta-learning algorithm [34], encompassing two primary phases: the task-level update and the global parameter update. For each task $T_{i} \in T^{t}$, we commence with the initial model parameters $\theta ^{*}$. Subsequently, we employ the gradient descent method to update these parameters, yielding the updated model parameters denoted as $\theta _{i}$. This process can be formulated as follows: 


(8) \begin{align*}& \theta_{i} = \theta^{*} - \alpha \nabla_{\theta} L_{i}(\theta^{*}),\end{align*}

where $L_{i}$ is the mean square error, $\alpha $ denotes the learning rate of the inner loop, and $\nabla _{\theta } L_{i}(\theta ^{*})$ symbolizes the gradient of the model parameters with respect to the loss function of task $T_{i}$.

In the global parameter update, we use the model parameters updated at the task level $\theta _{i}$ derived from the inner loop updates of all tasks to update the global model parameters $\theta ^{*}$. Specifically, we compute the average difference between the model parameters $\theta _{i}$ of all tasks and the current global model parameters $\theta ^{*}$, then add this average difference to the current global model parameters $\theta ^{*}$, yielding the updated global model parameters $\theta ^{*}$. This mechanism is mathematically encapsulated as follows: 


(9) \begin{align*}&\theta^{*} = \theta^{*} + \beta \left(\frac{1}{K}\right) \sum_{i} (\theta_{i} - \theta^{*}),\end{align*}

where $\beta $ signifies the magnitude of the global parameter update, and $K$ is the total number of tasks involved in the global parameter update.

Through an iterative process, we will refine the model parameters, culminating in optimal initial parameters $\theta $. This initial configuration empowers MetaFluAD to rapidly adapt to the specific influenza subtype or lineage despite having limited data while simultaneously mitigating overfitting.

In the meta-testing phase, MetaFluAD leverages a subset of known antigenic distances from $T^{s}$ to fine-tune the model to further specialize in the specific characteristics and relationships within $T^{s}$. During this process, MetaFluAD adjusts the learned parameters $\theta $ from the meta-training stage, and obtains the task-specific parameters $\theta ^{\prime}$ specifically tailored for the target dataset $T^{s}$. Now, antigenic distances in the target influenza subtype can be predicted using this model.

## Results and discussion

### Implementation

To evaluate the proposed method, we carry out experiments on five datasets of influenza viruses: D_H3N2, D_H1N1, D_H5N1, D_Yam, and D_Vic. These evaluations aim at investigating the generalizability and adaptability of MetaFluAD when applied to diverse influenza subtypes. Specifically, each experiment designates one dataset as meta-test set, representing a specific virus subtype. Meanwhile, the remaining datasets consisting of different subtypes serve as the meta-training sets, providing opportunities for the model to learn shared patterns and variations across multiple virus subtypes or lineages. More details of meta-learning process is explained in Supplementary Text S2.

The models’ performance is evaluated using three metrics: Mean Squared Error (MSE), Mean Absolute Error (MAE), and the $R^{2}$ score. The detailed calculation formulas are provided in Supplementary Text S3.

### Performance evaluation

To empirically assess the superiority of MetaFluAD, we employed multiple quantitative prediction models as baselines. These baselines include Lees [14], Liao [8], Yiao [12], and a GRU-based approach, as well as various network embedding methods like Node2Vec, LINE, Attri2Vec, and AANE [22]. Detailed descriptions of all models and implementation details are provided in the Supplementary Text S4 and S5.

Moreover, a five-fold cross-validation method was employed to assess the models. And, to eliminate variability, the study conducted 10 independent experiments using different random seeds, calculating the average of all metrics as a comprehensive measure of the model’s performance on specific target tasks. This method aims to provide a more robust performance evaluation framework, ensuring that the proposed model demonstrates high generalization capabilities and robustness across a wide range of tasks.

**Figure 2:**
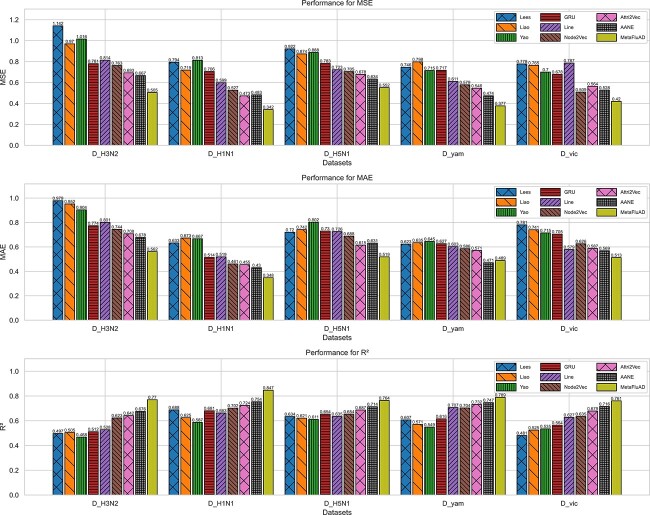
The performance (MSE, MAE, and $R^{2}$) of MetaFluAD and various baseline models on different datasets.

For MetaFluAD, the initial parameters obtained after each iteration were fine-tuned on the test data. The results for each dataset were recorded separately and compared with those of the baseline models. The statistical results are presented in Fig. 2. Additionally, Supplementary Table S4 and Figure S1 present the model’s average performance across all datasets.

The experimental results above show that MetaFluAD consistently outperforms the best baseline models across all datasets. This confirms that MetaFluAD can effectively learn strain representations within a unified space that encompasses both antigenic and genetic features. For the baseline models, traditional machine learning methods like Liao [8], Lees [14], and Yiao [12] rely heavily on hand-crafted features, leading to limited performance in predicting antigenic distances. While simple deep learning models like the GRU-based method directly extract features from HA1 sequences, their accuracy remains modest. Network embedding approaches like AANE [22] leverage both node attributes and topology, further boosting performance. However, their effectiveness falls short of MetaFluAD, which leverages a GNN-based encoder in conjunction with a powerful meta-learning framework, learning comprehensive strain representations within a unified space encompassing both antigenic and genetic features. Furthermore, meta-learning enables knowledge transfer across different influenza subtypes, allowing MetaFluAD to predict antigenic distances with limited data. By merging the power of a GNN-based encoder and meta-learning, MetaFluAD offers a promising approach for accurate antigenic distance prediction in influenza viruses. This novel method holds significant potential to contribute to developing effective vaccines and efficiently monitoring viral evolution.

Five-fold cross-validation yielded Supplementary Figures S2 and S3, which further substantiate MetaFluAD’s robustness. Supplementary Figure S2 presents a scatter plot of the model’s predicted values against the actual values, while Supplementary Figure S3 depicts the density distribution of prediction residuals. These figures reveal a linear relationship between the predicted values and the actual antigen distances, alongside a narrow central peak in the error distribution. These observations indicate that MetaFluAD’s predictions exhibit high accuracy and consistency.

To further validate the effectiveness of the initialization parameters learned through meta-learning, we compared the performance of MetaFluAD and the baseline model when fine-tuned with varying amounts of data. We designed three sets of experiments, employing 5%, 10%, and 20% of the data, respectively, for parameter fine-tuning in the meta-testing phase. The average results of 10 independent experiments are shown in Table 1, Supplementary Table S1 and S2, respectively. Across all three adaptive scenarios, our proposed MetaFluAD demonstrated a significant improvement over other baselines in terms of MSE, MAE, and $R^{2}$ metrics. The results indicate that even under data-limited conditions, the initialization parameters obtained through meta-learning enable MetaFluAD to perform exceptionally well, highlighting its advantages and practicality in scenarios with restricted data.

**Table 1:** Comparison of MetaFluAD and the baseline model performance with 5% of the data

		Methods								
Dataset	Metric	Lees	Liao	Yao	GRU	LINE	N2V	A2V	AANE	MetaFluAD
D_H3N2	MSE	1.366	1.418	1.476	1.473	1.409	1.397	1.297	1.202	**0.860**
	MAE	1.306	1.303	1.343	1.213	1.195	1.134	1.388	1.131	**0.833**
	R$^{2}$	0.331	0.344	0.301	0.339	0.359	0.423	0.466	0.467	**0.574**
D_H1N1	MSE	1.176	1.278	1.302	1.381	1.290	1.252	1.134	1.118	**0.807**
	MAE	0.920	0.941	1.073	0.944	0.949	0.985	0.997	0.943	**0.718**
	R$^{2}$	0.412	0.421	0.381	0.469	0.422	0.469	0.442	0.451	**0.620**
D_H5N1	MSE	1.508	1.352	1.412	1.439	1.468	1.402	1.457	1.333	**0.907**
	MAE	1.108	1.176	1.143	1.081	1.117	1.108	1.175	1.093	**0.794**
	R$^{2}$	0.349	0.403	0.367	0.414	0.427	0.413	0.453	0.464	**0.589**
D_yam	MSE	1.341	1.178	1.247	1.196	1.122	1.207	1.186	1.102	**0.718**
	MAE	1.030	0.982	1.012	0.937	1.116	1.048	0.927	0.977	**0.771**
	R$^{2}$	0.371	0.467	0.393	0.384	0.387	0.411	0.471	0.462	**0.602**
D_vic	MSE	1.383	1.298	1.319	1.216	1.144	1.189	1.306	1.172	**0.773**
	MAE	1.178	1.108	1.221	1.117	1.203	1.027	1.115	1.018	**0.764**
	R$^{2}$	0.335	0.347	0.309	0.371	0.410	0.302	0.434	0.454	**0.501**

Note: “N2V” represents Node2Vec, and “A2V” represents Attri2Vec. The best scores are marked in bold and the second-best scores are marked underline.

**Figure 3:**
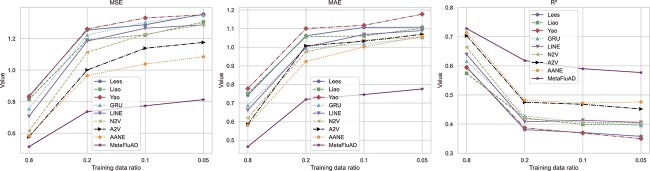
Average performance evaluation across five datasets: the graphs depict the average performance metrics of MetaFluAD compared with several baseline models, trained with different data ratios of 80%, 20%, 10%, and 5%.

Additionally, Fig. 3 illustrates the comparative average performance metrics of MetaFluAD and various baseline models across five datasets. Each model was trained using different subsets of data: 80%, 20%, 10%, and 5%. Notably, our method, MetaFluAD, consistently exhibits superior performance across varying data volumes. Furthermore, although the performance of each baseline model significantly declines with reduced data volumes, the performance degradation of MetaFluAD is more stable, demonstrating its robustness.

### Ablation study

#### Effect of meta learning

In order to evaluate the effect of meta-learning-based parameter initialization on the performance of MetaFluAD, we compare MetaFluAD and FluAD (without meta-learning initialization) on D_H3N2, D_H1N1, D_H5N1, D_Vic, and D_Yam.

**Figure 4:**
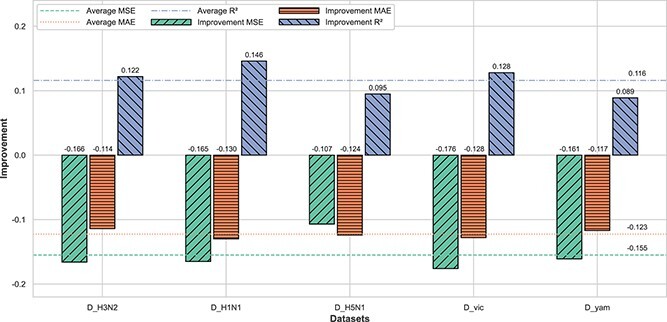
The gains of meta-learning parameter initialization in MetaFluAD across D_H3N2, D_H1N1, D_H5N1, D_Vic, and D_Yam datasets; the heights of the bars represent the difference in the performance of MetaFluAD on three metrics when initiated with and without meta-learning; the higher bars indicate a greater improvement due to meta-learning initialization; the straight line represents the average performance improvement of this metric on each dataset.

Figure 4 demonstrates consistent performance improvements across various datasets with parameter initialization via meta-learning in MetaFluAD. This strategy results in notable gains of 0.089–0.146 in $R^{2}$, along with reductions of 0.114–0.130 in MAE and reductions of 0.107–0.176 in MSE. These results demonstrate that incorporating meta-learning for parameter initialization in MetaFluAD leads to improved performance when compared with MetaFluAD without this method. This suggests that the meta-learning method enables the MetaFluAD model to effectively transfer knowledge from one subtype or lineage to another, thereby mitigating the challenges posed by limited data availability. MetaFluAD, equipped with meta-learning initialization, generalizes better and performs more robustly against various influenza subtypes.

#### Effect of the blocks of MetaFluAD

We investigated the impact of combining GNN embedding modules with self-attention mechanisms on the MetaFluAD model’s ability to predict antigenic distances. Ablation experiments were conducted using five-fold cross-validation, averaged across multiple datasets, to assess the performance of MetaFluAD compared with three variant models. These variants were constructed by removing specific components from the GAT and self-attention mechanisms. The results, as shown in Supplementary Table S3, indicate that MetaFluAD, when fully integrating both GNN embedding and self-attention modules, significantly improves prediction performance, thus validating the effectiveness of this combined approach.

### The antigenic cartography

**Figure 5:**
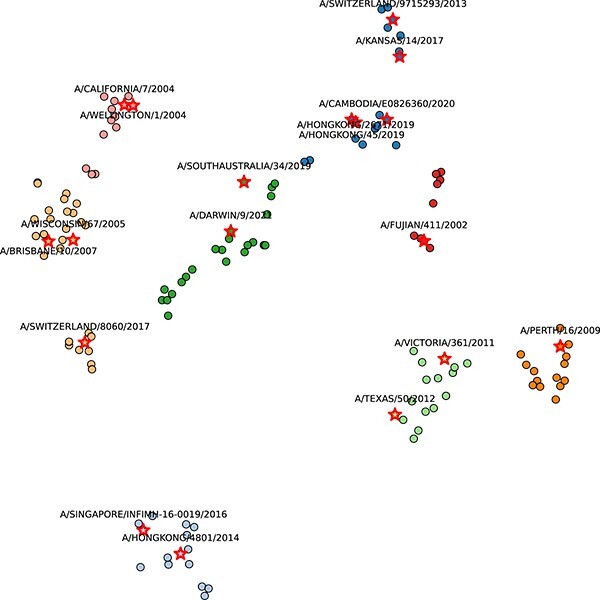
Antigenic cartography of D_H3N2 using t-SNE dimensionality reduction on strain vectors generated by MetaFluAD; the strain colors denote distinct antigenic clusters determined by k-means clustering; the annual WHO-recommended vaccine strains are highlighted with red pentacles and labeled.

To further analyze the effect of MetaFluAD’s antigenic distance predictions, we projected the embedding vectors of strains in the D_H3N2 dataset, generated by MetaFluAD, onto a two-dimensional plane (Fig. 5). This visualization, known as antigenic cartography, allows us to explore the relationships between influenza strains based on their antigenic distances and identify potential clusters or patterns that could inform influenza prediction and vaccine development.

Within the generated antigenic cartography (Fig. 5), clusters of strains with similar antigenic properties can be observed, indicating potential connections between them. One observation is that the recommended vaccine strain consistently resides within the same antigenic cluster as the dominant reference strain at the time of selection. For instance, A/Fujian/411/2002, the vaccine strain recommended for the 2004 influenza season in October 2003, belonged to the same antigenic cluster as reference strains A/Finland/170/2003, and A/Wyoming/3/2003. Similarly, A/Texas/50/2012, recommended for the 2014 influenza season in October 2013, belongs to the same antigenic cluster as A/Hong Kong/146/2013, A/Stockholm/1/2013, further solidifying the link between vaccine strain selection and antigenic clustering.

**Figure 6:**
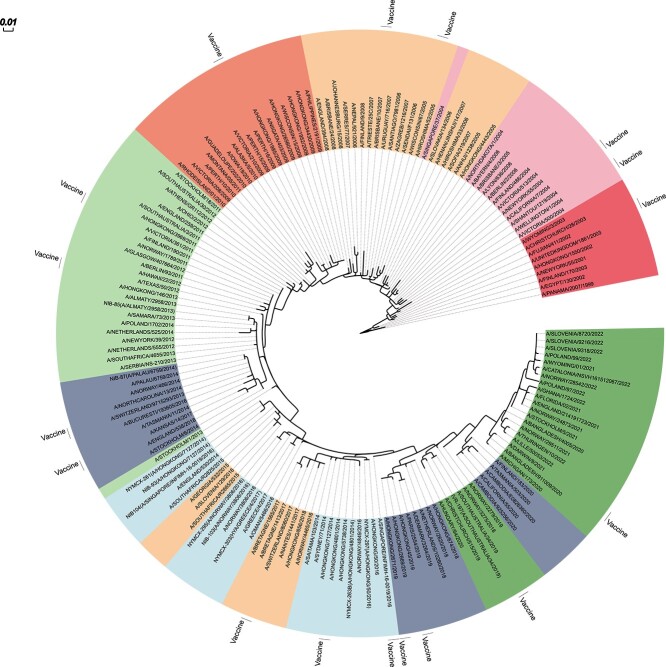
A maximum likelihood circular phylogenetic tree of the HA1 in D_H3N2, and color-coded based on antigenic clusters from Fig. 5, was created using Evolview v3; recommended vaccines are labeled with the text ‘Vaccine’.

To further elucidate the relationship between genetic relatedness and antigenic properties, we construct a maximum likelihood phylogenetic tree of the HA1 in D_H3N2 (Fig. 6) using MEGA 11 [26]. While phylogenetic trees provide valuable insights into the genetic evolution of viruses, they do not directly capture antigenic changes, which are crucial for understanding antigenic drift. We then employ Evolview v3 [35] to color-code the tree based on the antigenic clusters of Fig. 5. The color coding reveals a notable correspondence between the phylogenetic tree and antigenic mapping. Closely related HA1 sequences typically occupy the same antigenic cluster, in line with the observations of Smith *et al*. [2]. Although antigenic clusters appear cohesive on the phylogenetic tree, relying only on genetic data is insufficient for the determination of antigenic clusters.

Despite sharing an antigenic cluster with most reference strains of the 2003 influenza season, A/Fujian/411/2002 diverged significantly from reference strains in the subsequent 2004–2005 season. This antigenic difference suggests a potential decline in its cross-protective efficacy against dominant strains during that period. This hypothesis aligns with observations made by Belongia *et al*. [36], who reported a notable reduction in vaccine effectiveness against the 2004–2005 flu season, potentially attributable to the antigenic mismatch between the vaccine and circulating viruses. Similarly, despite apparent genetic and antigenic compatibility with the reference strains during the 2013–2014 influenza season, vaccine strain A/Texas/50/2012 proved ineffective for the 2014–2015 season due to substantial antigenic drift [37, 38]. This can be evidenced by the fact that A/Texas/50/2012 resides within a distinct antigenic cluster, diverging from the reference strains prominent during the 2014–2015 influenza season, such as A/England/530/2014 and A/GEORGIA/532/2015. In contrast, A/Perth/16/2009, recommended as a vaccine for the 2010–2011 influenza season, falls into the same antigenic cluster as the reference strains in 2010. A/Victoria/361/2011, recommended for multiple seasons (2012–2013, 2013, 2013–2014), also shares the same antigenic cluster as most of the reference strains from 2012 to 2014. This aligns with the high vaccine effectiveness during that period (https://www.cdc.gov/flu/vaccines-work/effectiveness-studies.htm).

Overall, MetaFluAD’s antigenic distance predictions and the utilization of antigenic cartography provide valuable insights into the relationships between influenza strains.

## Conclusion

While hemagglutination inhibition assays are the gold standard for detecting influenza antigenic variation, their high cost and restricted availability significantly hinder timely vaccine strain selection. This scarcity of HI data hinders global surveillance and our ability to respond quickly to emerging influenza threats. To address this challenge, we propose a novel meta-learning approach that utilizes knowledge transfer across different influenza subtypes or lineages to predict antigenic distances with limited data. Our method significantly decreased the prediction MSE compared with previous approaches, and demonstrated its effectiveness in antigenic cartography. The ability to predict antigenic distances makes MetaFluAD a valuable tool for early-stage vaccine development workflows, facilitating informed decision-making. Furthermore, the strain vectors obtained from MetaFluAD offer direct insights into dominant antigenic clusters in influenza viruses, enabling the rapid detection of antigenic drift. This supports timely updates to vaccine formulations, enhancing their efficacy and improving public health responses to influenza outbreaks.

Currently, the model is trained on limited data and focuses only on reference strains. Future research could expand this framework by incorporating test strains and enlarging the dataset, potentially enabling more accurate predictions. Additionally, the functionality and usability of the developed web server need to be extended to better serve the research community.

**Key Points**

- We proposed a comprehensive framework for knowledge transfer across influenza subtypes or lineages.
- MetaFluAD accurately predicts quantitative antigenic distances in scenarios with limited data by utilizing meta-learning techniques.
- Utilizing a GNN-based encoder within a robust meta-learning framework, MetaFluAD integrates antigenic and genetic data, achieving comprehensive strain representations.
- The strain vectors obtained from MetaFluAD offer direct insights into dominant antigenic clusters in influenza viruses, facilitating the rapid detection of antigenic drift.
- Compared with other existing methods, MetaFluAD demonstrates high accuracy and robustness across various influenza subtypes or lineages.

## Supplementary Material

MetaFluAD_Supplementary_Data_bbae395

## Data Availability

The data and source code are available at https://github.com/kpollop/metafluad. A web server is available at http://47.120.16.183:7860/.
