# Supplementary material for: MetaFluAD: meta-learning for predicting antigenic distances among influenza viruses
Source: Brief Bioinform. 2024 Aug 12;25(5):bbae395. doi: 10.1093/bib/bbae395 (PMC11317534; doi:10.1093/bib/bbae395)
Supplement: MetaFluAD_Supplementary_Data_bbae395 [file metafluad_supplementary_data_bbae395.pdf]

Supplementary Data for

MetaFluAD: meta-learning for predicting antigenic distances of influenza viruses

Qitao Jia, Yuanling Xia, Fanglin Dong and Weihua Li

Weihua Li
liweihua@ynu.edu.cn

This PDF file includes:

Supporting text
Figures S1 to S3
Tables S1 to S4
SI References

Supporting Information Text

Text S1: Algorithm.

Algorithm 1 Construction of Antigenic Dissimilarity Network

Input: Set of n strains $\mathcal{V} = \{v1, v2, \ldots, vn\}$, the HA1 sequence characterization of each strain
Output: Antigenic dissimilarity network $\mathcal{G} = (\mathcal{V}, \mathcal{X}, \mathcal{E})$
Initialize the set of edges $\mathcal{E} = \{\}$
Initialize the attribute matrix $\mathcal{X} = []$
Initialize the adjacency matrix A of size $n \times n$
for each strain $v_i \in \mathcal{V}$ **do**
    Protvec process the HA1 sequence of v_i into a feature vector x_i
    Add x_i to the attribute matrix $\mathcal{X}$
    for each strain $v_j \in \mathcal{V}$, $v_j \neq v_i$ **do**
        Calculate distance d_{ij} between v_i and v_j
        Add edge (v_i, v_j) to $\mathcal{E}$
        Update adjacency matrix $A[i, j] = A[j, i] = d_{ij}$
    end for
end for
Form the antigenic dissimilarity network $\mathcal{G} = (\mathcal{V}, \mathcal{X}, \mathcal{E})$ using $\mathcal{V}$, $\mathcal{X}$, and $\mathcal{E}$
Return $\mathcal{G}$

Text S2: Experimental Design. Given that there are five datasets, the paper performs five separate sets of experiments. The experimental setup for each set of experiments is as follows:

- **Experiment 1:**
  - **Meta-Test Set:** D_H3N2, representing the A/H3N2 subtype.
  - **Meta-Training Sets:** D_H1N1, D_H5N1, D_Yam, and D_Vic, representing A/H1N1, A/H5N1, B/Victoria, and B/Yamagata, respectively.
- **Experiment 2:**
  - **Meta-Test Set:** D_H1N1, representing the A/H1N1 subtype.
  - **Meta-Training Sets:** D_H3N2, D_H5N1, D_Yam, and D_Vic, representing A/H3N2, A/H5N1, B/Victoria, and B/Yamagata, respectively.
- **Experiment 3:**
  - **Meta-Test Set:** D_H5N1, representing the A/H5N1 subtype.
  - **Meta-Training Sets:** D_H3N2, D_H1N1, D_Yam, and D_Vic, representing A/H3N2, A/H1N1, B/Victoria, and B/Yamagata, respectively.
- **Experiment 4:**
  - **Meta-Test Set:** D_Yam, representing the B/Yamagata lineage.
  - **Meta-Training Sets:** D_H3N2, D_H1N1, D_H5N1, and D_Vic, representing A/H3N2, A/H1N1, A/H5N1, and B/Victoria, respectively.
- **Experiment 5:**
  - **Meta-Test Set:** D_Vic, representing the B/Victoria lineage.
  - **Meta-Training Sets:** D_H3N2, D_H1N1, D_H5N1, and D_Yam, representing A/H3N2, A/H1N1, A/H5N1, and B/Yamagata, respectively.

For each experiment, the model undergoes training using the meta-training sets to learn the shared patterns and variations across the different influenza subtypes. After training, the model's performance is evaluated on the designated meta-test set to assess its generalizability and adaptability to the specific influenza subtype or lineage.

Text S3 : Evaluation Metrics. The model's performance is evaluated using three metrics: Mean Squared Error (MSE), Mean Absolute Error (MAE), and R^2 score.

$$\mathrm{MSE} = \frac{1}{n}\sum_{i=1}^{n}(y_i - \hat{y}_i)^2 \quad [1]$$

$$\mathrm{MAE} = \frac{1}{n}\sum_{i=1}^{n}|y_i - \hat{y}_i| \quad [2]$$

$$R^2 = 1 - \frac{\sum_{i=1}^{n}(y_i - \hat{y}_i)^2}{\sum_{i=1}^{n}(y_i - \bar{y})^2} \quad [3]$$

where n is the total number of samples, $\bar{y}$ is the average of all actual antigenic distance values, y_i is the i-th actual antigenic distance value, and $\hat{y}_i$ is the model's predicted antigenic distance value for the i-th value. The smaller the MAE and MSE, the higher the prediction accuracy of the model. R^2 focuses on the accuracy of the model prediction. If R^2 is close to 1, it indicates that the model's prediction is very good.

Text S4 : baselines.

- Lees (1), Liao (2), and Yao (3) all used the original paper method to process the HA1 sequence, employing linear regression, multiple regression, and random forest as explained in their respective papers.

- GRU (4), a variant of recurrent neural networks, is employed to learn patterns and features within the HA sequence for antigenic distance prediction, utilizing a three-layer GRU network.

- Node2Vec (5), a Skip-Gram-based algorithm, generates node sequences through random walks. The random walk length is 4, each node performs 8 random walks, and the parameters are set to $p = 1$ and $q = 0.8$. The remaining parameters use default settings.

- LINE (6) builds the network using antigenic distances, with a breadth-first search restricting node connections to two hops. It models and merges first and second-order proximities of each node based on scenario needs. The negative sampling parameter is set to 4, and the other parameters use the default.

- Attri2Vec (7) maps the strain sequence representations of nodes to a new feature space using a transformation matrix, effectively capturing the relationships between strains. This method optimizes the feature vectors obtained from the mapping by maximizing the dot product between the representations of nodes and their respective neighbors, thereby enhancing the model's accuracy in predicting biological characteristics.

- AANE (8), using weighted attribute network embedding, generates node representations based on sequence features, with antigenic distances serving as edge weights.

Text S5: Implementation Details. The experimental code is implemented using the open-source machine learning framework PyTorch (https://pytorch.org). The GCN encoders are implemented with the open-source geometric deep learning extension library, PyTorch Geometric (https://github.com/pyg-team/pytorch_geometric). All experiments are accelerated using an NVIDIA GeForce RTX 3090 graphics card.

Table S1. Performance comparison of metaFluAD and baseline models using 10% of the data

		Methods								
Dataset	Metric	Lees	Liao	Yao	GRU	LINE	N2V	A2V	AANE	MetaFluAD
D_H3N2	MSE	1.359	1.378	1.478	1.333	1.394	1.307	1.184	<u>1.052</u>	**0.831**
	MAE	1.318	1.243	1.279	1.183	<u>1.095</u>	1.194	1.245	1.112	**0.813**
	R^2	0.336	0.341	0.319	0.374	0.369	0.403	0.475	<u>0.481</u>	**0.602**
D_H1N1	MSE	1.096	1.116	1.311	1.287	1.280	1.099	<u>0.964</u>	0.978	**0.757**
	MAE	0.918	0.947	1.011	0.925	0.935	0.951	0.898	<u>0.837</u>	**0.691**
	R^2	0.430	0.491	0.411	0.468	0.436	0.459	0.452	<u>0.464</u>	**0.621**
D_H5N1	MSE	1.411	1.297	1.392	1.428	1.450	1.372	1.327	<u>1.173</u>	**0.842**
	MAE	1.105	1.133	1.094	1.016	1.041	1.088	1.115	<u>1.001</u>	**0.762**
	R^2	0.378	0.419	0.393	0.419	0.457	0.393	0.473	<u>0.479</u>	**0.611**
D_yam	MSE	1.295	1.093	1.197	1.196	1.122	1.207	1.034	<u>1.001</u>	**0.695**
	MAE	0.988	0.971	0.999	0.937	1.096	1.028	<u>0.921</u>	0.932	**0.729**
	R^2	0.378	0.389	0.405	0.382	0.401	0.446	0.472	<u>0.478</u>	**0.592**
D_vic	MSE	1.279	1.238	1.279	1.273	1.082	1.149	1.155	<u>1.014</u>	**0.747**
	MAE	1.174	1.013	1.205	1.109	1.094	<u>1.001</u>	1.028	1.059	**0.734**
	R^2	0.336	0.356	0.315	0.389	0.401	0.337	0.444	<u>0.469</u>	**0.526**

Note: "N2V" represents Node2Vec, and "A2V" represents Attri2Vec. The best scores are marked in bold, and the second-best scores are underlined.

Table S2. Performance comparison of metaFluAD and baseline models using 20% of the data

		Methods								
Dataset	Metric	Lees	Liao	Yao	GRU	LINE	N2V	A2V	AANE	MetaFluAD
D_H3N2	MSE	1.368	1.334	1.421	1.134	1.226	1.101	0.982	<u>0.963</u>	**0.789**
	MAE	1.309	1.251	1.249	1.164	1.004	<u>0.991</u>	1.046	1.054	**0.792**
	R^2	0.342	0.358	0.335	0.379	0.366	0.411	0.485	<u>0.492</u>	**0.619**
D_H1N1	MSE	1.087	1.091	1.216	1.205	1.121	0.948	0.887	<u>0.848</u>	**0.711**
	MAE	0.917	0.939	0.974	0.907	0.947	0.939	0.872	<u>0.806</u>	**0.678**
	R^2	0.429	0.466	0.418	0.476	0.452	0.474	<u>0.484</u>	0.483	**0.645**
D_H5N1	MSE	1.341	1.289	1.308	1.357	1.447	1.273	1.203	<u>1.156</u>	**0.816**
	MAE	1.021	1.109	1.103	0.996	1.031	1.052	1.112	<u>0.979</u>	**0.731**
	R^2	0.386	0.415	0.390	0.424	0.454	0.412	<u>0.494</u>	0.477	**0.644**
D_yam	MSE	1.201	1.096	1.155	1.161	1.109	1.114	0.878	<u>0.874</u>	**0.674**
	MAE	0.981	0.966	1.006	0.895	1.066	0.981	<u>0.861</u>	0.905	**0.681**
	R^2	0.402	0.484	0.413	0.405	0.405	0.460	<u>0.491</u>	0.476	**0.615**
D_vic	MSE	1.274	1.154	1.208	1.262	1.017	1.131	1.054	<u>0.984</u>	**0.697**
	MAE	1.082	1.021	1.197	1.098	0.980	0.998	0.955	<u>0.931</u>	**0.716**
	R^2	0.347	0.354	0.326	0.400	0.417	0.332	0.439	<u>0.485</u>	**0.568**

Note: "N2V" represents Node2Vec, and "A2V" represents Attri2Vec. The best scores are marked in bold, and the second-best scores are underlined.

Table S3. Results of ablation experiments removing the GNN module and self-attention module, respectively.

Model	MSE	MAE	R^2
MetaFluAD-G-T	0.721	0.684	0.641
MetaFluAD-G	0.637	0.634	0.688
MetaFluAD-T	0.595	0.627	0.704
MetaFluAD	**0.439** (↓ 0.282)	**0.485** (↓ 0.199)	**0.786** (↑ 0.145)

Note: "MetaFluAD-G-T" represents MetaFluAD without both the GNN module and the self-attention module. "MetaFluAD-G" represents MetaFluAD without the GNN module. "MetaFluAD-T" represents MetaFluAD without the self-attention module. The best scores are marked in bold, with the improvement/decrease in performance indicated in parentheses.

Table S4. Average performance metrics for each model using 80% of the data for training

model	MSE	MAE	R^2
Lees	0.8764	0.7472	0.5814
Liao	0.8252	0.7486	0.5694
Yao	0.8264	0.7466	0.5500
GRU	0.7330	0.6700	0.6054
Line	0.7068	0.6456	0.6320
Node2Vec	0.6166	0.6210	0.6636
Attri2Vec	0.5908	0.5872	0.6928
AANE	0.5572	0.5558	0.7214
MetaFluAD	**0.4392**	**0.4852**	**0.7862**

Note: The table shows the average prediction metrics across different datasets for the models when trained using 80% of the data.

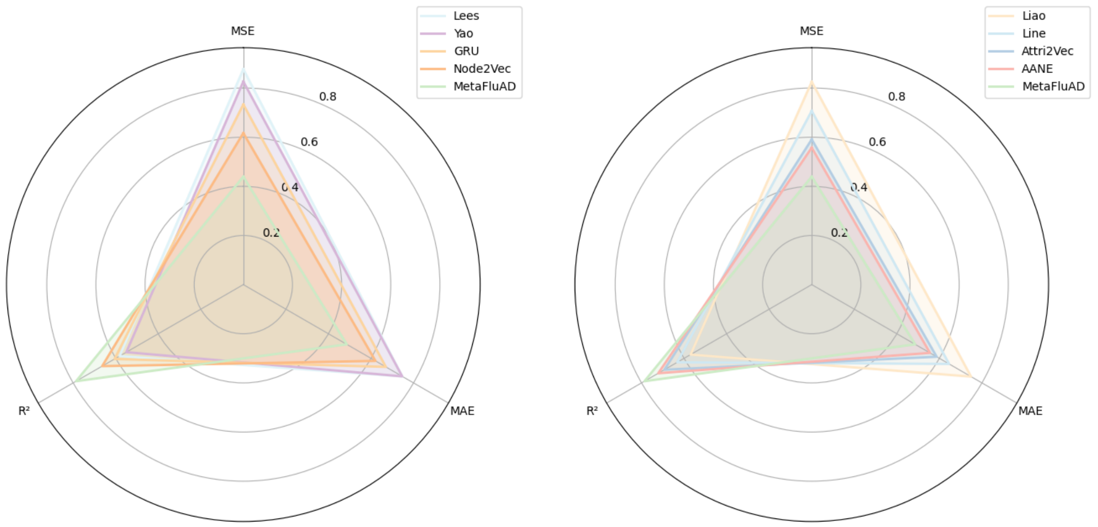

Figure S1. Comparison of average performance metrics between MetaFluAD and existing methods across various datasets. Each dataset was split with 80% of the data used for training and 20% for evaluation. MetaFluAD consistently outperforms existing methods, demonstrating superior predictive accuracy and robustness.

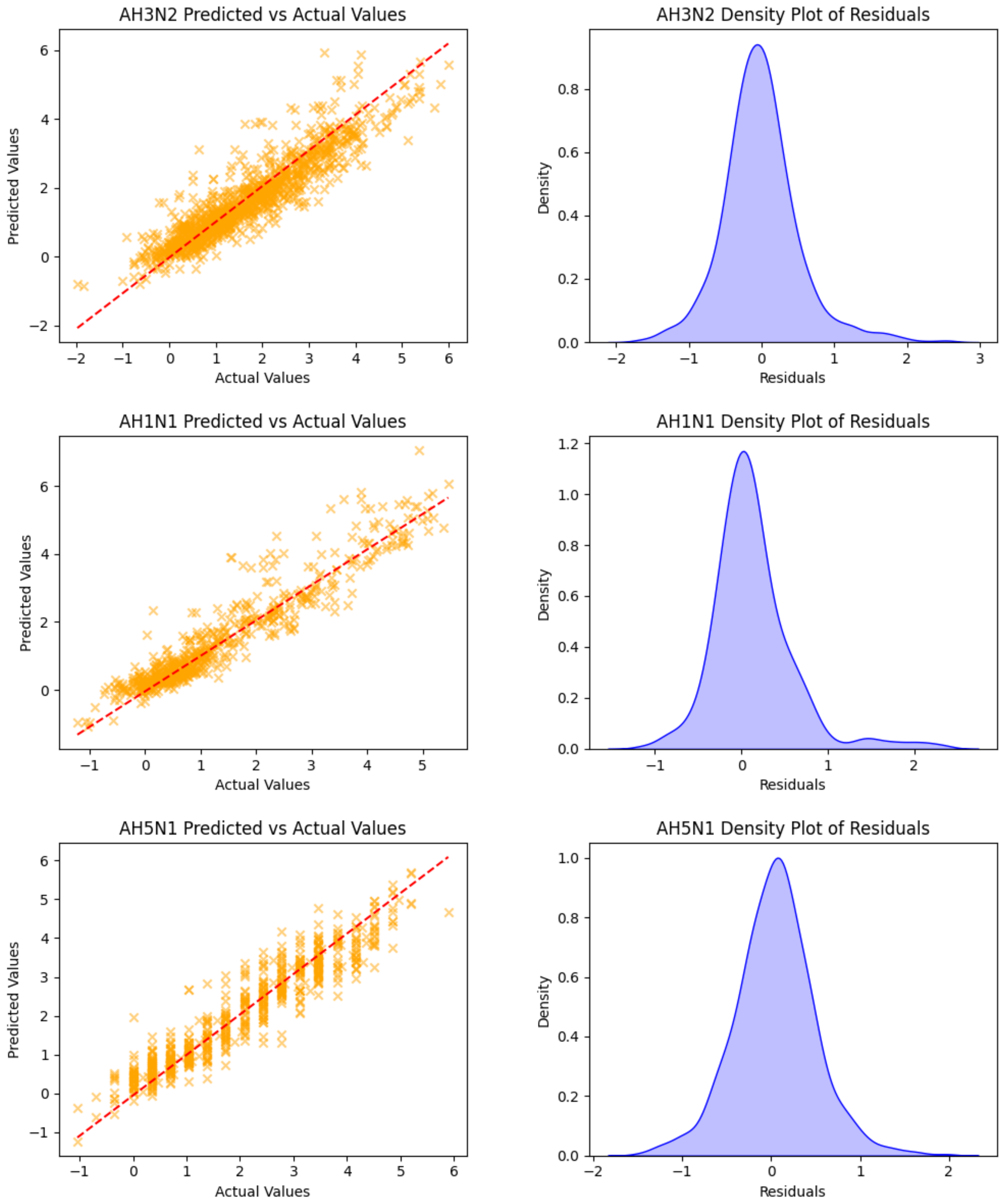

Figure S2. Left panels: Scatter plots comparing model predictions against actual values, with a red dashed line at the 45° diagonal indicating perfect prediction. Right panels: Density plots of prediction residuals, illustrating the distribution of errors. A narrower, symmetrical distribution centered around zero indicates a more accurate model with consistent predictive performance. Each dataset was split with 80% of the data used for training and 20% for evaluation.

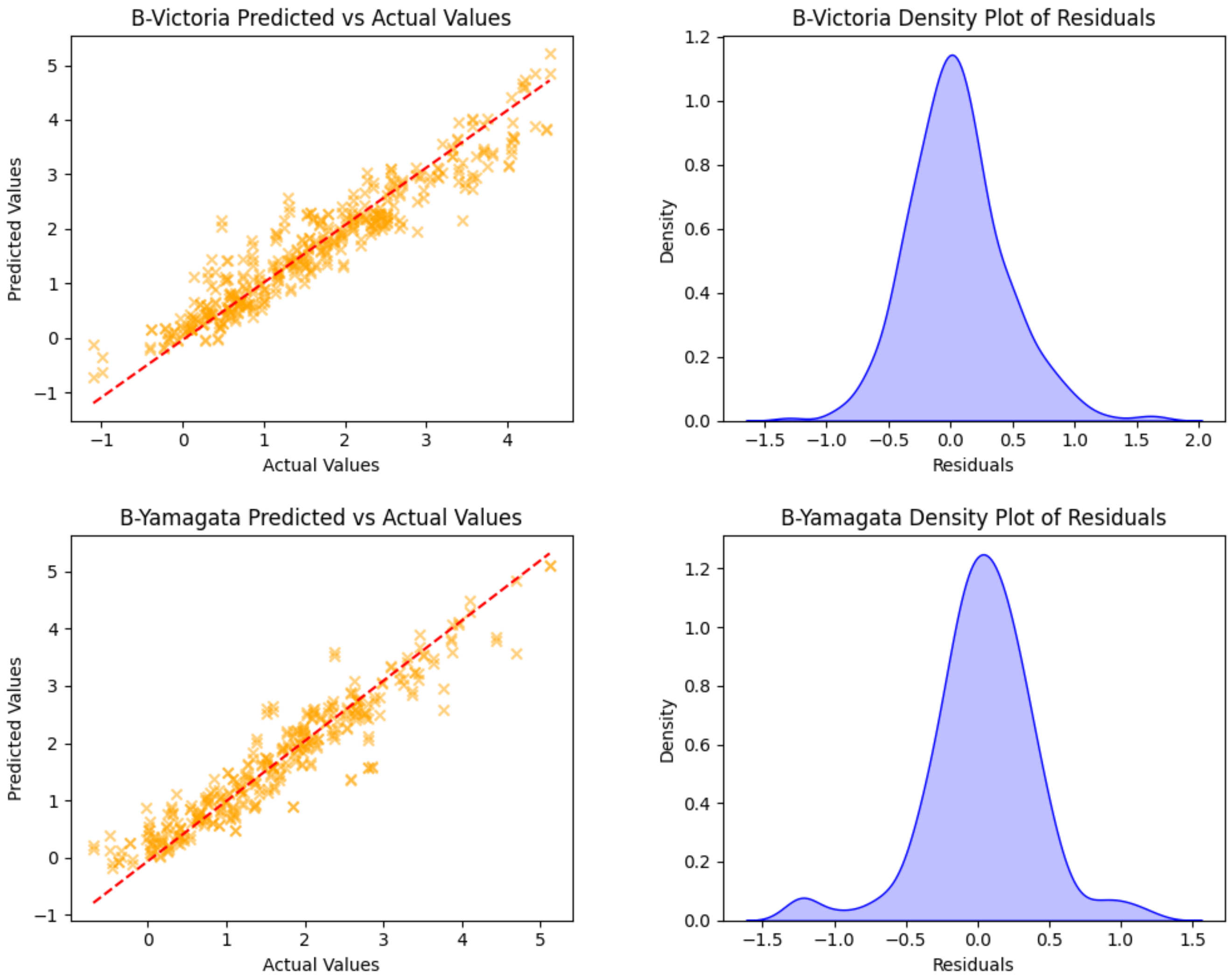

Figure S3. Left panels: Scatter plots comparing model predictions against actual values, with a red dashed line at the 45° diagonal indicating perfect prediction. Right panels: Density plots of prediction residuals, illustrating the distribution of errors. A narrower, symmetrical distribution centered around zero indicates a more accurate model with consistent predictive performance. Each dataset was split with 80% of the data used for training and 20% for evaluation.

References

1. WD Lees, DS Moss, AJ Shepherd, A computational analysis of the antigenic properties of haemagglutinin in influenza a h3n2. *Bioinformatics* **26**, 1403–1408 (2010).
2. YC Liao, MS Lee, CY Ko, CA Hsiung, Bioinformatics models for predicting antigenic variants of influenza A/H3N2 virus. *Bioinformatics* **24**, 505–512 (2008).
3. Y Yao, et al., Predicting influenza antigenicity from hemagglutintin sequence data based on a joint random forest method. *Sci. reports* **7**, 1545 (2017).
4. K Cho, B Van Merriënboer, D Bahdanau, Y Bengio, On the properties of neural machine translation: Encoder-decoder approaches. *arXiv preprint arXiv:1409.1259* (2014).
5. A Grover, J Leskovec, node2vec: Scalable feature learning for networks in *Proceedings of the 22nd ACM SIGKDD international conference on Knowledge discovery and data mining.* pp. 855–864 (2016).
6. J Tang, et al., Line: Large-scale information network embedding in *Proceedings of the 24th international conference on world wide web.* pp. 1067–1077 (2015).
7. D Zhang, J Yin, X Zhu, C Zhang, Attributed network embedding via subspace discovery. *Data Min. Knowl. Discov.* **33**, 1953–1980 (2019).
8. F Peng, Y Xia, W Li, Prediction of antigenic distance in influenza A using attribute network embedding. *Viruses* **15**, 1478 (2023).
